# Irisin and myostatin serum levels in patients with established rheumatoid arthritis: correlation with radiographic progression and lean body mass

**DOI:** 10.1186/s42358-025-00505-z

**Published:** 2026-04-07

**Authors:** Jordana Miranda de Souza Silva, Rafaela Cavalheiro do Espírito Santo, Deborah Negrão Gonçalo Dias, Nayara Felicidade Tomaz Braz, Erica Leandro Marciano Vieira, Eduarda Correa Freitas, Rafael Mendonça da Silva Chakr, Adriana Maria Kakehasi, Ricardo Machado Xavier

**Affiliations:** 1 Laboratory of Autoimmune Diseases, Rheumatology Department, Clinical Hospital of Porto Alegre, Porto Alegre, Brazil; 2 Health Research and Innovation Science Centre – Klaipeda University, Klaipeda, Lithuania; 3 Clinical Hospital of Federal University of Paraná, Curitiba, Brazil; 4 Interdisciplinary Medical Research Laboratory of the Faculty of Medicine, Federal University of Minas Gerais, Belo Horizonte, Brazil; 5 Centre for Addiction and Mental Health – CAMH and Department of Psychiatry University of Toronto, Toronto, Canada; 6 Postgraduate Program in Medicine: Medical Sciences, Federal University of Rio Grande do Sul, Porto Alegre, Brazil; 7 Rheumatology Unit, Clinic Hospital, Federal University of Minas Gerais, Belo Horizonte, Brazil

**Keywords:** Rheumatoid arthritis, Myostatin, Irisin

## Abstract

**Background:**

The myokines irisin and myostatin participate in bone and skeletal muscle homeostasis and may characterize the clinical status of these tissues. The study aimed to evaluate the association of myokines serum levels with one-year radiographic progression and lean mass in individuals with rheumatoid arthritis (RA).

**Methods:**

Forty female individuals with RA, aged ≥ 18 years who met 2010 American College of Rheumatology criteria, and 30 individuals without RA and any chronical disease, matched by sex and body mass index (BMI) were included. Serum levels of irisin and myostatin were determined by immune assay. RA subjects had their radiographs of hands and feet evaluated by Sharp/van der Heijde score (SHS) at two timepoints, baseline and after one year. At baseline, disease activity was calculated by Disease Activity Score 28-C reactive protein (DAS28-CRP), body composition was evaluated using dual X-ray absorptiometry (DXA), muscle strength was assessed by handgrip test and chair rising test (CRT), and physical function was assessed by Health Assessment Questionnaire-Disability Index (HAQ-DI) and timed up and go (TUG) test.

**Results:**

Mean age of individuals was 56 ± 7.8 years, mean DAS28-CRP was 3.3 ± 1.3, mean disease duration was 11.2 ± 9.2 years, and mean BMI was 28.1 ± 5.1 kg/m^2^. Rapid radiographic progression and low lean mass were present in 17.5% and 14.8% of the RA individuals, respectively, and showed no correlation with irisin and myostatin. Myostatin was significantly lower in RA than in controls (3021.7 ± 1217.2 vs. 4049.0 ± 1610.0 pg/ml; *p* = 0.011), and individuals treated with biologic disease-modifying antirheumatic drugs (bDMARDs) showed higher irisin levels than individuals non-treated with bDMARDs (31.7 ± 7.6 vs. 25.7 ± 6.8 ng/ml; *p* = 0.033). RA duration was correlated with baseline SHS (*r* = 0.563; *p* = 0.001) and appendicular lean mass index (ALMI; *r*= -0.451; *p* = 0.004), and irisin levels were positively correlated with TUG (*r* = 0.338; *p* = 0.35) in RA.

**Conclusions:**

Long-term RA was related with higher SHS and lower ALMI, and the late disease stage of included individuals possibly masked the association of myokines with one-year radiographic progression or low lean mass. Otherwise, bDMARDs treatment influenced myokines circulating levels, specifically irisin.

## Background

Rheumatoid arthritis (RA) is a systemic inflammatory disease characterized by chronic synovitis that leads to cartilage and bone destruction [1]. Erosive joint damage is a hallmark of RA and over time causes deformity and contributes to disability in patients [2]. The progression of joint erosion is variable among individuals with RA and is usually detectable as radiographic damage in affected joints [3]. In addition to joint involvement, about 31% of the individuals with RA are affected by sarcopenia, and frequently present lower muscle mass, strength, and physical performance than their general counterparts [4–7]. Despite current treatment strategies, aiming the early and rapid decrease of disease activity, radiographic progression still occurs in a significant proportion of individuals, and the loss of muscle mass is not always addressed in RA treatment [8, 9].

Several skeletal muscle-derived humoral cytokines and growth factors, collectively termed myokines, which include irisin and myostatin, have recently been implicated not only in muscle but also in bone physiological and pathological metabolism [10]. Myokines are expressed in skeletal muscle, which is also their main target tissue, although irisin and myostatin are also active to a considerable extent in bone [11, 12]. Myostatin is a crucial myokine, described as potent suppressor of skeletal muscle mass growth and development, upregulating proteolysis pathways and inhibiting muscle protein synthesis and regeneration [13]. Myostatin also acts as an inhibitor of bone mass and remodeling and, specifically in arthritis, increases osteoclast formation and bone destruction [12]. Notably, the myostatin expression undergoes opposite direction to irisin, which functions as a pro-myogenic factor and enhances muscle protein synthesis, in addition to playing a role in bone mass and strength gain by osteoblastogenesis stimulation [14–16].

The intimate relationship between skeletal muscle and bone led to the concept of the “bone-muscle unit”, partly because muscle and bone mass and strength are positively affected by physical exercise, while both are compromised by ageing and situations of disuse [17]. Thus, myokines may establish a communication network between the two tissues, in addition to the mechanotransduction. Low muscle area, density and strength has been reported in RA subjects, and over time the worsening of skeletal muscle density has been correlated to higher disease activity [6, 18]. Additionally, muscle deficits have been shown to contribute to inferior cortical bone structure and trabecular bone mineral density in RA, which was speculated to be related the loss of mechanical loading [19]. As skeletal muscle and bone are affected in RA, and both are sources of myokines, it is possible that the levels of these markers may correlate with clinical outcomes.

The identification of individuals at risk of greater radiographic damage and significant muscle loss is critical to adapt the treatment and prevent disease progression, which stresses the need for biomarkers predicting structural joint and skeletal muscle changes in individuals with RA [20, 21]. Thus, this study aimed to address the associations of baseline serum irisin and myostatin concentrations with lean mass and one-year radiographic progression in RA patients. Secondarily, association of serum myokines with treatment and physical function was also assessed.

## Materials and methods

### Subjects

Forty female individuals with RA attending the Rheumatology Division at Clinical Hospital of Porto Alegre, Brazil, who fulfilled the America College of Rheumatology/European League Against Rheumatism 2010 criteria, were enrolled in the study [22]. Thirty individuals without RA and any chronical disease, matched by sex and body mass index (BMI) were enrolled as controls for the evaluation of myokines serum levels at baseline. RA individuals’ radiographs of hands and feet were evaluated at two timepoints, baseline and after one year. RA individuals’ disease activity, body composition, physical function, and Erythrocyte Sedimentation Rate (ESR) and C-Reactive Protein (CRP) were also evaluated at baseline. Information on the therapeutic regimens used by patients for the treatment of RA was obtained from hospital medical records. The study was conducted in accordance with the ethical standards of the Declaration of Helsinki and was approved by the Hospital Ethics Committee (approval number: CEP 2018 − 0223). Written informed consent was obtained from all participants prior to their inclusion in the study.

### Radiographic progression

One-year progression of joint damage was assessed with the use of the van der Heijde modification of the total Sharp score (SHS) method [8]. Baseline radiographs of hands and feet were performed ± 3 months from the blood collection visit. The scoring was performed by a single experienced reader blinded to patient characteristics and aware of image sequence. The intrareader variability described by the intraclass correlation coefficient (ICC) was 0.92 for the radiographic progression rate. Among the 40 individuals with RA completing the study, 37 had evaluable x-rays from both timepoints, baseline and one-year.

### Body composition

Subjects underwent total body dual-emission x-ray absorptiometry (DXA) scanning using a Hologic densitometer (Delphi Systems, Hologic, Inc., Bedford, MA) to measure appendicular lean mass and total fat mass. Body composition estimates were adjusted for height^2^ to generate appendicular lean mass index (ALMI, kg/m^2^) and fat mass index (FMI, kg/m^2^). Additionally, fat-adjusted ALMI (ALMI_FMI_) Z-Scores were generated, based on residuals obtained from the regression of ALMI Z-Score on FMI Z-Score within age, sex, and race categories. Thus, “low fat-adjusted lean for age” was defined as ALMI_FMI_ Z-score ≤ -1 [21].

### Muscle strength

Muscle strength was assessed by handgrip test and chair rising test (CRT). The CRT is a timed test of lower limb muscle strength evaluation. The participant was asked to stand up and sit down from a chair five times in a row, as fast as possible, without using the arms [23]. The handgrip strength describes the power or strength of the hand muscles used to grasp or grip, and its evaluation was performed using a Jamar Hydraulic Hand Dynamometer. The participant was instructed to squeeze the equipment as hard as possible for 5 s for the maximal isometric voluntary contraction of each hand to be quantified.

### Physical function

Disability was assessed by the Health Assessment Questionnaire-Disability Index (HAQ-DI), which ranges from 0.0 to 3.0 and higher values ​​represent worst functional status [24]. In the timed up and go (TUG) test, an individual sit on a standard height armchair with his hands placed on the armrest. The person is asked to stand up (using the arms), walk 3 m at a normal speed, turn around, return to the chair, and sit down again. The more time needed to complete the test, the greater the restriction of mobility and the higher the risk of falling [25].

### Laboratory assessments

Fasting blood sample was collected in the morning, from the ulnar vein of the participants, in vacuum tube by a qualified healthcare professional, complying with the norms for the use of sharp instruments in an aseptic environment. The blood was processed within 2 h to obtain the serum (3000 x g, 4 °C, 15 min). The serum was aliquoted and stored at -80 °C until the analysis. Enzyme-linked immunosorbent assay (ELISA) was performed to evaluate irisin (Phoenix Pharmaceuticals, CA, USA) and myostatin (R&D Systems, Minneapolis, MN, USA) serum levels according to manufacturer’s instructions. Clinical laboratory assays [Erythrocyte Sedimentation Rate (ESR) and C-Reactive Protein (CRP)] were performed using standard methods in Hospital Clinical Pathology Department.

### Statistical analysis

Data distribution was preliminarily checked for normality with the Kolmogorov-Smirnov test. According to number of groups, nonparametric tests Kruskal-Wallis test or Mann-Whitney U test were used. Data are presented as median (interquartile range). The Kruskal-Wallis test was utilized to compare the difference of myokines serum levels and ALMI among radiographic progression and DAS28-CRP categories. The Mann-Whitney U test was used to compare the difference of myokines serum levels between patients and controls, between patients using or not using biological disease-modifying antirheumatic drugs (bDMARDs), and between ALMI_FMI_ categories. Spearman’s correlation analysis was used to analyze the correlation among myokines serum levels and mean SHS score, ALMI, DAS28-CRP, HAQ-DI, ESR and CRP, and to analyze the correlation between disease duration and lean mass and Sharp score. P-values ≤ 0.05 were considered statistically significant. The Statistical Package for the Social Sciences (SPSS Inc., Chicago, IL, USA) version 20.0 was used to analyze the data.

## Results

### Subjects’ demographics and baseline characteristics

Table 1 presents the baseline characteristics of the women in RA and healthy control groups. RA individuals included in the study presented mean age of 56 years, mean disease duration of 11.2 years and mean BMI of 28.1 kg/m^2^. Control individuals presented mean age of 37 years and mean BMI of 26.1 kg/m^2^. Mean disease activity score was 3.3. Baseline SHS higher than 25 units was present by 43.2% of the RA individuals, while 17.5% were classified as having low fat-adjusted lean for age (ALMI_FMI_ Z-score ≤ -1). Thirteen RA individuals were being treated with bDMARDs: abatacept (*n* = 4), etanercept (*n* = 4), tocilizumab (*n* = 3), adalimumab (*n* = 1) and rituximab (*n* = 1) (Table 1).

**Table 1:** Demographic profile at baseline of female individuals with RA and control subjects

	RA individuals (*n* = 40)	Control individuals (*n* = 30)
Age in years (mean ± SD)	56.3 ± 7.8	37.4 ± 7.8
Irisin in ng/ml (median, IQR)	27.5 (23.5–31.8)	28.0 (23.9–33.1)
Myostatin in pg/ml (median, IQR)	3096.7 (1958.4-3903.1)	3743.5 (3043.3-4827.3)
BMI in kg/m^2^ (mean ± SD)	28.1 ± 5.1	26.1 ± 5.2
RA duration in years (mean ± SD)	11.2 ± 9.2	
Baseline SHS (median, IQR)	15.7 (17.5-46.15)	
DAS28-CRP score (median, IQR)	3.2 (2.7–3.8)	
bDMARDs, n (%)	13 (32.5)	
ALMI in kg/m^2^ (mean ± SD)	6.3 ± 0.7	
FMI in kg/m^2^ (mean ± SD)	11.9 ± 3.6	
ALMI_FMI_ Z-score (mean ± SD)	0.07 ± 1	
ALMI_FMI_ Z-score ≤ -1, n (%)	7 (17.5)	
HAQ score (median, IQR)	0.75 (0.69–1.2)	
CRT in seconds (mean ± SD)	17.5 ± 12.2	
TUG in seconds (median, IQR)	9.4 (9.0-10.7)	
Handgrip strength in kg (median, IQR)	9.0 (7.1-12-7)	
ESR in mm/h (median, IQR)	29.0 (25.9–39.1)	
CRP in mg/dl (median, IQR)	5.0 (5.7–14.1)	

SD, standard deviation; IQR, interquartile range; RA, rheumatoid arthritis; SHS, Sharp van der Heijde Score; DAS28-CRP, disease activity score 28- C-reactive protein; bDMARDs, biological disease-modifying antirheumatic drugs; BMI, body mass index; ALMI, appendicular lean mass index; FMI, fat mass index; ALMI_FMI_ Z-score, appendicular lean mass index adjusted by fat mass index Z-score; HAQ, health assessment questionnaire; CRT, chair rising test; TUG, timed up and go test; ESR, erythrocyte sedimentation rate; CRP, C-reactive protein

### Myokines analysis

**Fig. 1:**
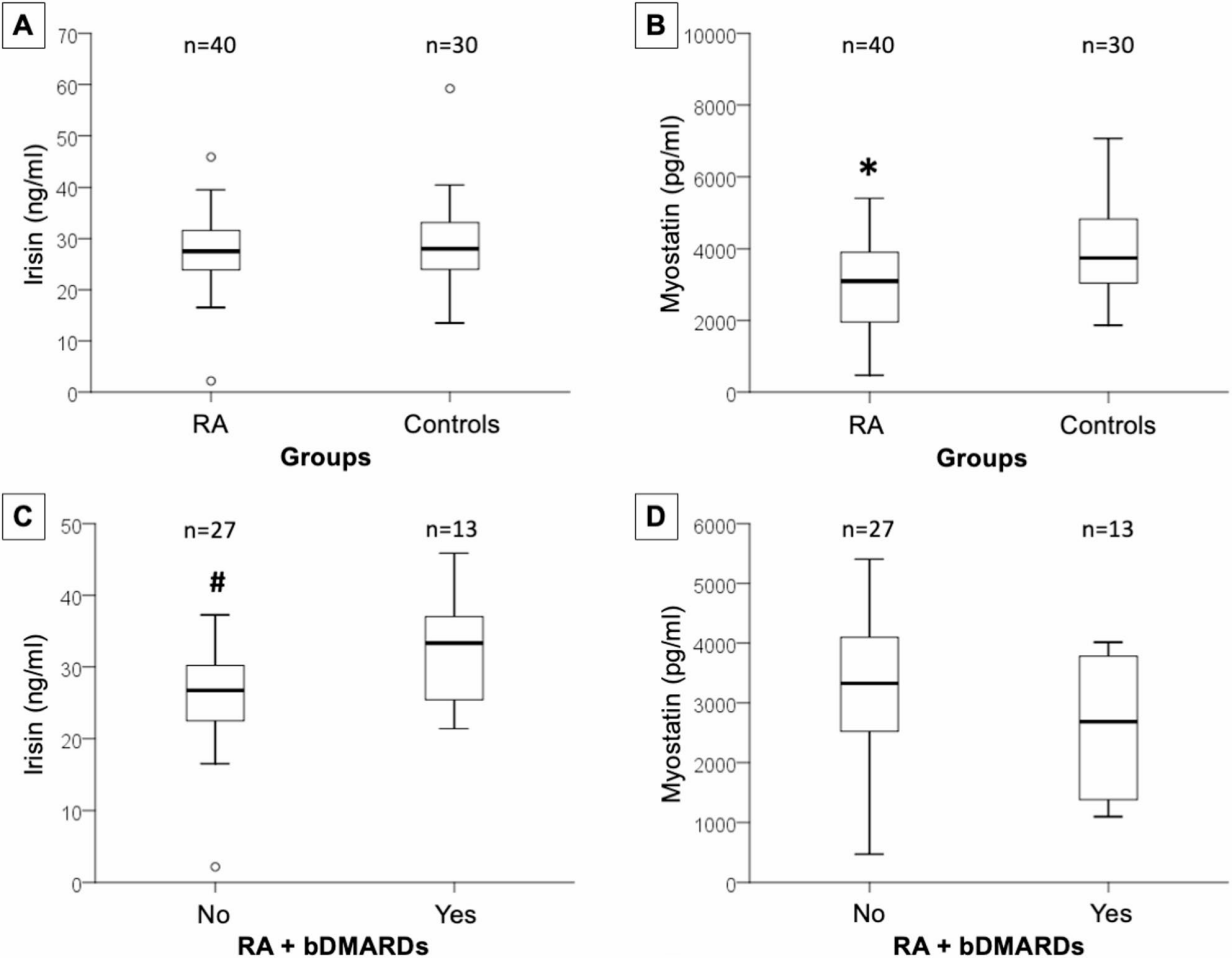
Irisin (**A**) and myostatin (**B**) levels of RA and control individuals. Irisin (**C**) and myostatin (**D**) levels individuals with RA treated with bDMARDs and non-treated with bDMARDs. Data were analyzed by Mann-Whitney U test and are represented by median (interquartile range). *p ≤ 0,05 RA vs. controls. #*p* ≤ 0,05 RA treated with bDMARDs vs. RA non-treated with bDMARDs

Myostatin levels were significantly lower in RA individuals than in control subjects (3096.7 (1958.4-3903.1) vs. 3743.5 (3043.3-4827.3) pg/ml; *p* = 0.011), while irisin levels did not differ between RA and control groups (27.5 (23.5–31.8) vs. 28.0 (23.9–33.1) ng/ml; *p* = 0.590) (Fig. 1A and B). RA individuals treated with bDMARDs presented irisin levels significantly higher than individuals non-treated with bDMARDs (33.3 (25.4–37.0) vs. 26.7 (22.5–30.2) ng/ml; *p* = 0.033). Myostatin levels tended to be lower in RA individuals treated with bDMARDs, compared to non-treated individuals (2683.2 (1381.7-3781.8) vs. 3325.5 (2520.1-4098.5) pg/ml; *p* = 0.144) (Fig. 1C and D). ESR and the levels of CRP were not correlated with irisin and myostatin serum levels.

### Radiographic progression

Irisin and myostatin levels were not associated with mean baseline and one-year SHS (*r*=-0.06, *p* = 0.68 and *r* = 0.01, *p* = 0.95). Irisin and myostatin levels did not differ when RA individuals were grouped into categories of radiographic progression: non-progressor (Δ = 0), slow progressor (Δ 0 ≤ 5) or rapid progressor (Δ > 5) (Table 2).

**Table 2:** Serum Irisin and myostatin levels according to Sharp Van der Heijde score categories in RA individuals

	*n*	Irisin (ng/ml)	Myostatin(pg/ml)	*p*
Baseline SHS				
0	1	45.9	3543.5	-
0.1-5	11	29.9 (25.4–31.3)	2940.5 (1381.7-3341.3)	ns
5.1–25	9	25.7 (24.5–33.3)	3753.1 (1958.4-4015.1)	ns
> 25	16	27.3 (20.7–31.3)	3461.2 (2242.2-4098.5)	ns
Progression				
Δ = 0	4	30.3 (24.9–38.3)	3153.3 (2445.4-3442.4)	ns
Δ 0 ≤ 5	28	26.1 (23.5–31.3)	3588.0 (1958.4-4098.5)	ns
Δ > 5	5	31.8 (28.4–37.0)	2242.2 (1892.5-3212.4)	ns

Values are expressed as the median (interquartile range). Data were analyzed by Kruskal-Wallis test. ns, non-significant. SHS, Sharp van der Heijde Score

### Lean mass

**Fig. 2:**
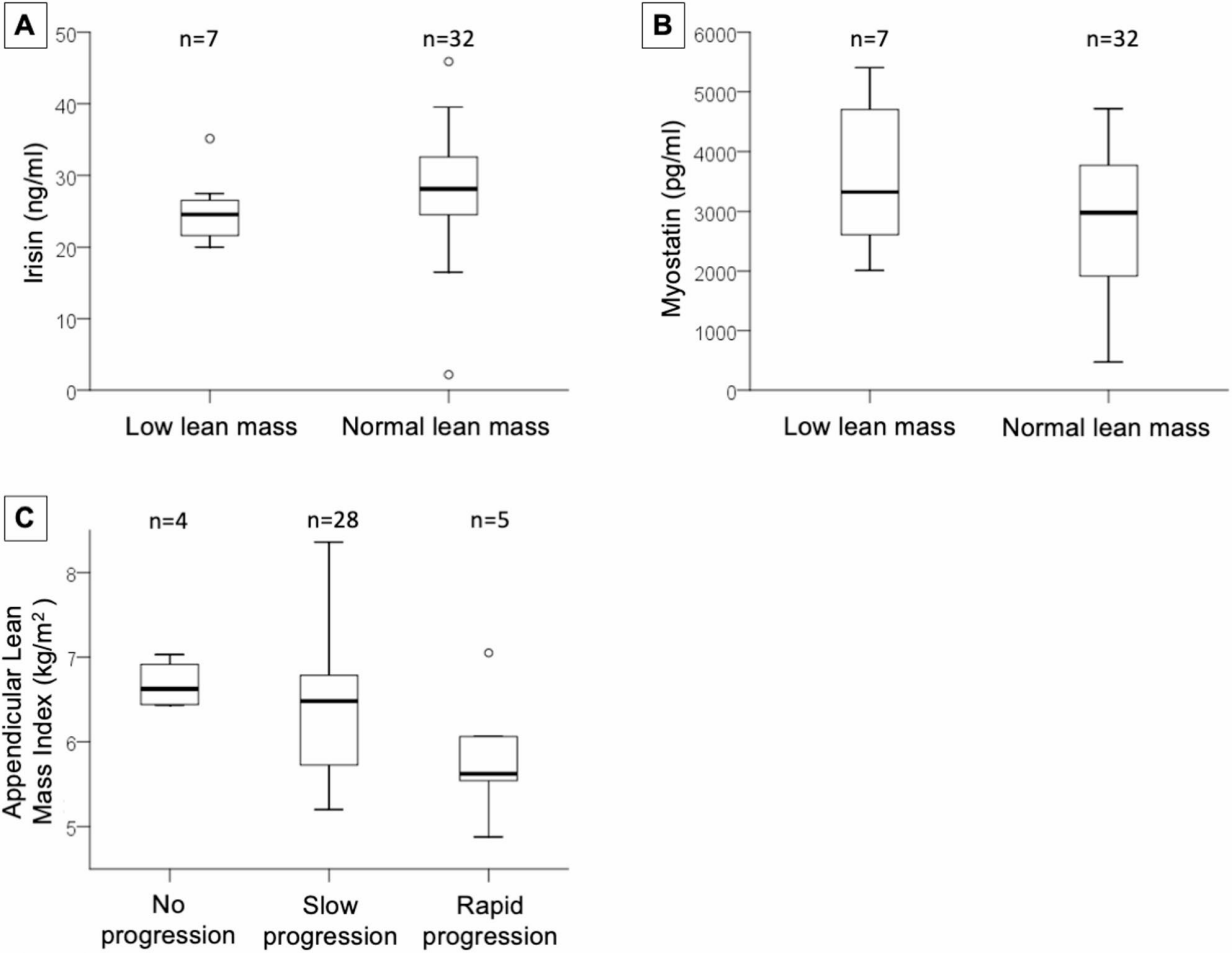
Irisin (**A**) and myostatin (**B**) levels of RA individuals with low lean mass and normal lean mass. Appendicular lean mass index of RA individuals with no progression, slow progression, and rapid progression (**C**). Data were analyzed by Mann-Whitney U test or Kruskal-Wallis test and are represented by median (interquartile range)

When RA individuals were grouped into low fat-adjusted lean for age (ALMI_FMI_ Z-score ≤ -1) or normal fat-adjusted lean for age (ALMI_FMI_ Z-score >-1) categories, no differences in irisin (24.5 (20.7–27.5) vs. 28.1 (24.5–32.6) ng/ml; *p* = 0.138) or myostatin (3325.5 (2242.2-5043.2) vs. 2977.8 (1907.5-3781.8) pg/ml; *p* = 0.181) levels were observed, respectively (Fig. 2A and B). ALMI was not significantly different among the categories of SHS radiographic progression (non-progression 6.6 (6.4–6.9); slow progression 6.4 (5.7–6.7); rapid progression 5.6 (5.5-6.0); *p* = 0.271).

### Physical function

No correlation was found among irisin and myostatin levels and CRT, handgrip strength and HAQ. Irisin serum levels were positively correlated with TUG (*r* = 0.339, *p* = 0.032), while myostatin showed no correlation.

### Disease activity and inflammatory markers

Irisin and myostatin were not associated with DAS28-CRP (*r* = 0.081; *p* = 0.630 and *r*= -0.041; *p* = 0.811) or different among the categories of disease activity severity (*p* = 0.147 and *p* = 0.601) (Table 3) [26]. The duration of the disease was correlated with baseline SHS (*r* = 0.563; *p* = 0.001) and ALMI (*r*= -0.451; *p* = 0.004).

**Table 3:** Serum Irisin and myostatin levels in RA individuals stratified by disease activity categories according to DAS28-CRP

	*n*	Irisin (ng/ml)	Myostatin(pg/ml)	*p*
DAS28-CRP				
Remission	11	26.1 (20.7–30.8)	3212.4 (1381.7-4285.3)	ns
Low	3	27.6 (22.5-35.15)	3325.5 (2977.8-4362.8)	ns
Moderate	13	26.4 (23.9–37.1)	3648.3 (2601.6-3898.4)	ns
High	11	29.9 (25.4–31.3)	2467.2 (1265.7-2965.4)	ns

Values are expressed as the median (interquartile range). Data were analyzed by Kruskal-Wallis test. ns, non-significant. DAS28-CRP, disease activity score 28-C reactive protein

## Discussion

The myokines irisin and myostatin participate in bone and skeletal muscle homeostasis and may be representative of the clinical status of these tissues. We found that levels of myostatin were diminished in individuals with RA but were not related to one-year radiographic progression and to lean mass content, as well as irisin. Patients using bDMARDs presented significantly higher serum irisin levels and a tendency toward lower myostatin concentrations compared to those not using bDMARDs, while disease duration was found to be correlated with baseline SHS and ALMI.

The individuals with RA included in our study were mostly slow progressors over one year. Although lower irisin and higher myostatin levels were expected in individuals with greater mean SHS or rapid radiographic progression, it was not observed in our study. Literature reports that irisin exerts anabolic action in bone, which is mediated primarily through the stimulation of bone formation [27]. In vivo administration of irisin increases cortical bone mass and strength, blunts the osteoblast differentiation inhibitor sclerostin, and modulates the Osteoprotegerin/RANKL to a physiological level, inhibiting osteoclasts formation and activity [11, 28]. On the other hand, myostatin has been shown to contribute to bone resorption in inflammatory arthritis. Specifically, it is highly expressed in the synovial tissue of RA patients and experimental arthritis models, where it promotes osteoclast differentiation [12]. Moreover, while Bialek et al. demonstrated that a soluble activin receptor (ActRIIB-Fc) enhances bone formation in mice, their findings suggest this effect may be mediated by inhibition of multiple ligands beyond myostatin [29]. Therefore, the role of myostatin in bone homeostasis may depend on the specific context and mechanism of inhibition. In our study, the absence of greater progression of joint erosion in one year may have influenced the power to detect correlations with the myokines. Also, the long disease duration and, consequently, the high SHS of the study participants could possibly mask the contribution of myostatin and irisin levels in RA bone erosion process. In fact, in RA individuals with low baseline SHS, higher myostatin levels were found, in comparison with healthy controls, and associated with a higher rate of radiographic progression [30]. However, another study identified lower myostatin serum levels in RA patients in remission compared to controls, as ours [31]. Although not significantly different, serum irisin levels tended to be lower in RA individuals in our study, similarly to a previous report, in which RA patients presented lower irisin levels than control subjects [32].

Skeletal muscle is admittedly the largest myokines secretor to the blood stream, and changes in their levels may reflect disorders in muscle tissue, mostly [33–36]. In our study, the individuals with RA with low lean mass were few and presented no significant difference in irisin and myostatin levels compared to the ones with normal lean mass. Previous data show that irisin induces muscle regeneration and hypertrophy by satellite cells activation and protein synthesis enhancing, in addition to preventing muscle atrophy by inhibiting ubiquitin-proteasome overactivity [14, 37]. Accordingly, low circulating irisin was reported to be associated with sarcopenia in postmenopausal women [33]. Otherwise, myostatin is known as an inhibitor of muscle growth, and its blockage by gene knockout, neutralizing antibody or soluble myostatin receptor, leads to an increase in muscle mass. Additionally, myostatin impairs muscle regeneration, downregulates muscle protein synthesis and stimulates proteolysis cascades. Although its serum levels are usually associated with negative muscle outcomes, such as sarcopenia, frailty, and reduced grip strength, there are also controversy findings [34–36]. In RA, serum myostatin was negatively correlated with skeletal muscle mass index (SMI) [38].We did not find associations between the myokines evaluated and the muscle mass of RA individuals, although 17.5% of them presented low lean mass. However, an additional factor contributing to myokines levels in RA may be the use of bDMARDs.

We identified significantly higher irisin levels in RA individuals treated with bDMARDs, while myostatin levels tended to be lower in RA individuals treated with bDMARDs, compared to non-treated ones. It has been demonstrated that the administration of tumor necrosis factor-α (TNF-α) to mice significantly reduced irisin gene and protein expression in skeletal muscle. Also, the treatment of myotubes with TNF-α and interleukin-1β (IL-1β) down-regulated irisin mRNA [39]. The RA systemic increase in pro-inflammatory cytokines possibly diminishes irisin secretion, as RA individuals present a tendency of lower irisin levels than controls in this study. Otherwise, the treatment with bDMARDs is likely to minimize the irisin reduction in individuals with RA. Positive feedback among pro-inflammatory cytokines (TNF-α and IL-1β) and myostatin have been shown in synovial fibroblasts isolated from RA patients [12, 40, 41]. Additionally, in culture of muscle cells, TNF-α promoted increase of myostatin expression through the NF-κB- pathway [42]. In a previous study reporting low myostatin levels in RA patients in remission, the authors argued that most of patients were using bDMARDs and possibly the immune modulation positively affected muscle mass, thereby decreasing myostatin [30]. Whereas, a cross-sectional study, in which only 6% of the RA patients were using bDMARDS, reported higher myostatin concentration in RA patients than in controls [38].

Associations between disease activity and irisin or myostatin serum levels were not identified in our study. In RA, negative correlation has been described between DAS28-ESR and serum irisin, while levels of myostatin have been associated with disease activity [32, 38, 43]. In osteoarthritis, otherwise, the serum and synovial concentrations of myostatin and irisin were equivalent and associated with higher and lower disease severity, respectively [44, 45]. Finally, the disease duration showed correlation with both ALMI and baseline SHS in individuals with RA. After the disease onset, patients may progressively present bone erosion, and reduction of leans mass as an extra-articular manifestation, but the irisin and myostatin serum levels are not representative of these bone and muscle alterations when the disease is established, possibly due to the modulation by bDMARDs use. The evaluation of physical function the RA individuals performed in our study demonstrated that irisin is positively associated with TUG, which assesses gait speed and lower extremity mobility and function, with higher values representing worse physical function. The association we found between irisin and TUG is not completely clear, since previous studies relate serum irisin with better physical function evaluated by grip strength [44, 46]. However, since irisin levels were significantly higher in patients using bDMARDs, this factor may have influenced the observed correlation between irisin levels and reduced gait speed.

The number of participants included is one of the limitations of this study, as well as the low proportion of RA individuals with greater radiographic progression and low lean mass. Reinforced by our results, established RA leads to cumulative erosions, joint space narrowing and extraarticular manifestations, including lower muscle mass, in addition to effects of the treatment. Our study highlights the associations of baseline serum irisin and myostatin with radiographic progression over one year and lean mass in RA patients, while also exploring their relationships with treatment and physical function. These findings offer novel insights into potential biomarkers of joint and muscle involvement in RA, contributing to a better understanding of disease pathophysiology and management.

## Conclusions

In our study, long-term RA was related with higher SHS and lower ALMI, while serum myokines were not associated with one-year radiographic progression or low lean mass. Also, the use of bDMARDs demonstrated to influence circulating levels of myokines, specifically irisin. In conclusion, the late disease of the included patients possibly masked the association of serum myokines with one-year radiographic progression or low lean mass in our study.

## Data Availability

The datasets used and/or analyzed during the current study are available from the corresponding author on reasonable request.

## Abbreviations

RA: Rheumatoid arthritis
ACR: American College of Rheumatology
BMI: Body mass index
ELISA: Enzyme-linked immunosorbent assay
SHS: Sharp/van der Heijde score
DAS28: Disease Activity Score 28
DXA: Dual X-ray absorptiometry
5XSST: The five Times Sit to Stand Test
HAQ-DI: Health Assessment Questionnaire-Disability Index
TUG: Timed up and go
ESR: Erythrocyte sedimentation rate
CRP: C reactive protein
bDMARDs: Biologic disease-modifying antirheumatic drugs
ALMI: Appendicular lean mass index
FMI: Fat mass index
SMI: Skeletal muscle mass index
TNF-α: Tumor necrosis factor-α
IL-1β: Interleukin-1β
